# Oyster Fermentation Broth Alleviated Tripterygium-Glycosides-Induced Reproductive Damage in Male Rats

**DOI:** 10.3390/molecules30173550

**Published:** 2025-08-29

**Authors:** Jiajia Yin, Hongguang Zhu, Yu Tian, Tengyu Ma, Wenjing Yan, Haixin Sun

**Affiliations:** College of Life Sciences, Qingdao University, Qingdao 266071, China; 15993607668@163.com (J.Y.); hongg_zhu@163.com (H.Z.);

**Keywords:** oyster, fermentation, tripterygium glycosides, sperm quality, oxidative stress, reproductive damage

## Abstract

In this study, oyster fermentation broth (OFB) was prepared by fermenting oysters with yeast, and its effects on oxidative stress and reproductive damage induced by tripterygium glycosides (TG) in male rats were investigated. Component analysis revealed that OFB contained bioactive substances including proteins (1.19 g/L), taurine (0.76 g/L), organic acids (2.30 mg/mL), polyphenols (123.00 mg GAE/L), flavonoids (1.97 mg RE/L), and zinc (1.10 mg/L). In vitro study revealed that OFB exhibited notable antioxidant activity, with a total antioxidant capacity of 1.28 U/mL, and DPPH, ABTS, and hydroxyl radical scavenging rates of 55.80%, 69.54%, and 48.36%, respectively. Animal experiments showed that, compared with the TG-induced model group, rats administered both low-dose (5 mL/kg) and high-dose (10 mL/kg) OFB showed significantly increased testis and seminal vesicle + prostate indices, sperm count, and serum testosterone (T) levels and decreased sperm malformation rate (*p* < 0.01 for all). Histological analysis of the testis revealed an increased number of spermatogenic cells and sperm within the seminiferous tubules, along with ameliorated pathological conditions compared to the model group. Potential mechanisms might be related to OFB increasing the activities of catalase (CAT), superoxide dismutase (SOD), and glutathione peroxidase (GSH-PX) enzymes and reducing levels of malondialdehyde (MDA) in testis (*p* < 0.01). The findings demonstrated that OFB successfully alleviated TG-induced reproductive damage in male rats, which might be attributed to its excellent antioxidant effect. The study offers valuable insights for producing functional foods from oysters and further validates OFB’s efficacy in promoting reproductive function.

## 1. Introduction

Oysters, as a typical marine resource with both medicinal and nutritional properties, are rich in proteins, amino acids, polysaccharides, lipids, and other nutrients [1]. They also contain highly bioactive functional components such as taurine, zinc, and selenium [2], which confer various biological activities, including antioxidant, anti-inflammatory, hepatoprotective, and reproductive-health-promoting effects [3,4,5]. Given their properties, oysters are ideal for use in functional food and beverage production. In recent years, a variety of oyster-based functional products, such as oyster peptide powders, oyster zinc tablets, and collagen oyster drinks, have emerged, demonstrating considerable market potential. However, most current products focus primarily on the extraction and application of specific bioactive substances from oysters, such as peptides and polysaccharides [6,7], while preparations of oyster fermented broth have received considerably less research attention.

Fermentation confers various benefits to food and is an effective approach for improving the nutritional composition and physicochemical properties of products [8]. Fermented products possess distinctive nutritional value and sensory attributes, making them highly popular worldwide. Yeast, an important microorganism widely utilized in the food, pharmaceutical, and bioenergy industries, includes common species such as *Saccharomyces cerevisiae*, *Pichia pastoris*, and *Rhodotorula* spp. Yeasts offer several advantages, including simultaneous substrate conversion, production of valuable metabolites, low cost, strong fermentation capacity, rapid growth, and adaptability to harsh environmental conditions [9,10,11]. Yeast fermentation offers significant advantages, including efficiency, safety, multifunctionality, and sustainability [12]. During the yeast fermentation process, the content of sugars or proteins in the fermentation substrate is reduced, resulting in the generation of beneficial molecules such as bioactive peptides, short-chain fatty acids, and polysaccharides. Additionally, the composition and content of phenolic compounds may also change [13,14]. Therefore, yeast fermentation not only alters the nutritional composition and proportions of the fermentation broth but also enhances its antioxidant capacity [15]. Among existing studies, microbial fermentation of oysters has largely centered around lactic acid bacteria [8,16], whereas research on the use of yeast for oyster fermentation is still relatively scarce. Consequently, utilizing yeast in oyster fermentation may open new avenues for marine product bioprocessing.

Globally, 8–12% of reproductive-aged couples experience fertility issues, nearly half of which stem from male-related factors [17]. Multiple factors contribute to male infertility, such as damage to the reproductive system, exposure to environmental pollutants, unhealthy living habits, and negative effects of certain medications [18,19,20]. Therefore, the development of functional foods or pharmaceuticals that can alleviate male reproductive impairment is urgently needed to help reduce the burden of reproductive dysfunction. Given that current studies on yeast-fermented oyster products primarily focus on the levels of active substances [21] and their general antioxidant and anti-inflammatory effects [22], investigations into their potential roles in testicular oxidative stress and male reproductive dysfunction remain limited. Further research in this area is thus warranted.

In the present study, *Saccharomyces cerevisiae* strain BV818 was employed to ferment oyster homogenate to prepare oyster fermentation broth (OFB). The changes in bioactive components in the fermentation mixture were subsequently analyzed. The bioactivities of OFB were evaluated in a male rat model of reproductive dysfunction. The findings may offer foundational support for the innovation and utilization of functional foods derived from oysters.

## 2. Results

### 2.1. Component Characterization and in Vitro Antioxidant Activity of OFB

#### 2.1.1. Characterization of Major Physicochemical Parameters and Bioactive Components in OFB

The changes in physicochemical parameters, minerals, organic acids, and amino acids before and after OFB fermentation are shown in Table 1. The experimental results showed that OFB contained 4.27 g/L of total acids, 2.54 g/L of reducing sugars, 1.19 g/L of proteins, 0.76 g/L of taurine, 121.10 mg/L of minerals, 2.30 mg/mL of organic acids, and 434.20 mg/L of free amino acids, suggesting the presence of functional bioactive components in OFB. Compared with the unfermented sample, the total acid content significantly increased (*p* < 0.05), while the levels of reducing sugars and proteins significantly decreased (*p* < 0.05) after yeast fermentation. Additionally, the total contents of minerals, organic acids, and free amino acids also increased. Detailed compositions of reducing sugars, minerals, organic acids, and free amino acids are presented in Table 1. The results revealed that the fermentation broth exhibited a high fructose/glucose ratio and contained abundant organic acids and sweet-tasting amino acids, which may be attributed to the sugars and precursor substances provided by honey as the fermentation substrate.

#### 2.1.2. Characterization of Phenolic Compounds and Antioxidant Activity of OFB

The phenolic profile and in vitro antioxidant activity of OFB before and after fermentation are shown in Table 2 and Table 3. After fermentation, OFB exhibited significantly increased total phenolic content (123.00 mg GAE/L), flavonoid content (1.97 mg RE/L), and antioxidant capacity (1.28 U/mL), along with higher DPPH, ABTS, and hydroxyl radical scavenging rates (55.80%, 69.54%, and 48.36%, respectively) (*p* < 0.05) (Table 2). A total of 11 phenolic compounds were identified before and after fermentation, and their chemical structures are shown in Figure S1. Before fermentation, the main compounds were quercetin, xanthurenic acid, caffeic acid, and clove3. After fermentation, quercetin, pyrogallol, salicylic acid, xanthurenic acid, 3,4-dihydroxyphenylpyruvic acid, 4-hydroxybenzaldehyde, naringenin, and caffeic acid were dominant. Fermentation notably altered their composition. These results align with previous findings [23], suggesting that fermentation can modulate the composition and content of substances in the fermentation broth and enhance its in vitro antioxidant capacity. The presence of quercetin, caffeic acid, robinin, and naringenin in the fermentation broth was mainly due to the addition of honey, while 3,4-dihydroxyphenylpyruvic acid and xanthurenic acid originated from yeast metabolism. Since different types of honey contain different phenolic profiles, they can affect the composition and levels of phenolics in the final product. These compound changes may be related to the substrates and the physiological activities of yeast, which utilized nutrients from oysters and honey for growth and metabolism, thereby promoting the release of active substances into the fermentation broth.

### 2.2. Growth Parameters of Rats

**Figure 1:**
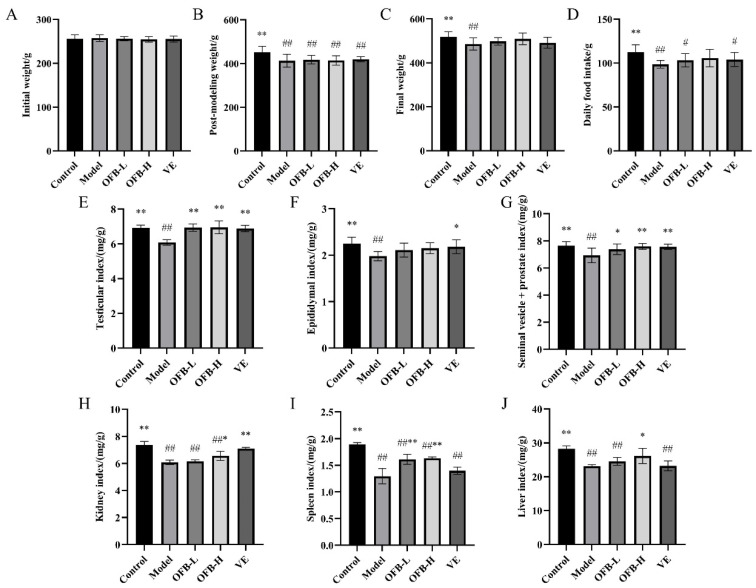
Comparison of growth parameters among groups of rats: (**A**) initial weight; (**B**) post-modeling weight; (**C**) final weight; (**D**) daily food intake; (**E**) testicular index; (**F**) epididymal index; (**G**) seminal vesicle + prostate index; (**H**) kidney index; (**I**) spleen index; (**J**) liver index. Symbol notation: # *p* < 0.05, ## *p* < 0.01 vs. control; * *p* < 0.05, ** *p* < 0.01 vs. model group.

As shown in Figure 1, the initial body weights did not differ significantly across all experimental groups. During the TG gavage period, rats in the control group remained in good condition, with natural weight gain, normal food and water intake, smooth and glossy fur, and active behavior. From the second week of TG administration, all groups except the control group exhibited soft, unformed stools, reduced activity, sparse, dry, and dull fur, as well as signs of hair loss. Following TG modeling, compared with the control group, the body weights in the model and experimental groups significantly decreased (*p* < 0.01). Following OFB gavage, compared with the control group, the model group showed significantly lower final body weight, food intake, testis index, epididymis index, seminal vesicle + prostate index, kidney index, spleen index, and liver index (*p* < 0.01), indicating that TG intake led to weight loss, reduced appetite, and organ atrophy in rats. Compared with the model group, the oyster fermentation broth low dose (OFB-L) and oyster fermentation broth high dose (OFB-H) groups exhibited significantly increased final body weight and food intake, as well as significantly elevated testis index, seminal vesicle + prostate index, and spleen index (*p* < 0.01). Wang et al. reported that LC-CS prepared from *Lycium chinense* and *Cuscutae semen* significantly improved the weights of testis and epididymis in a TG-induced reproductive dysfunction model [24], which is consistent with our findings. These results indicated that OFB intake alleviated anorexia and the reduction of organ indices caused by TG. Notably, the recovery effects of OFB on the testis and spleen indices even exceeded those observed in the VE group.

### 2.3. Hematological Parameters

The results are shown in Table 4. Compared with the control group, the LYM and NEU levels in the model group exhibited a significant decrease (*p* < 0.05), indicating that TG intake induced oxidative stress damage. After OFB gavage administration, the RBC and LYM levels in both the OFB-L and OFB-H groups were significantly increased (*p* < 0.01) compared with the model group, suggesting that OFB intake alleviated TG-induced damage. Additionally, changes in RBC, HGB, and LYM levels indicated that OFB provided even better recovery of hematological parameters than the VE group.

### 2.4. Sperm Quality Analysis

**Figure 2:**
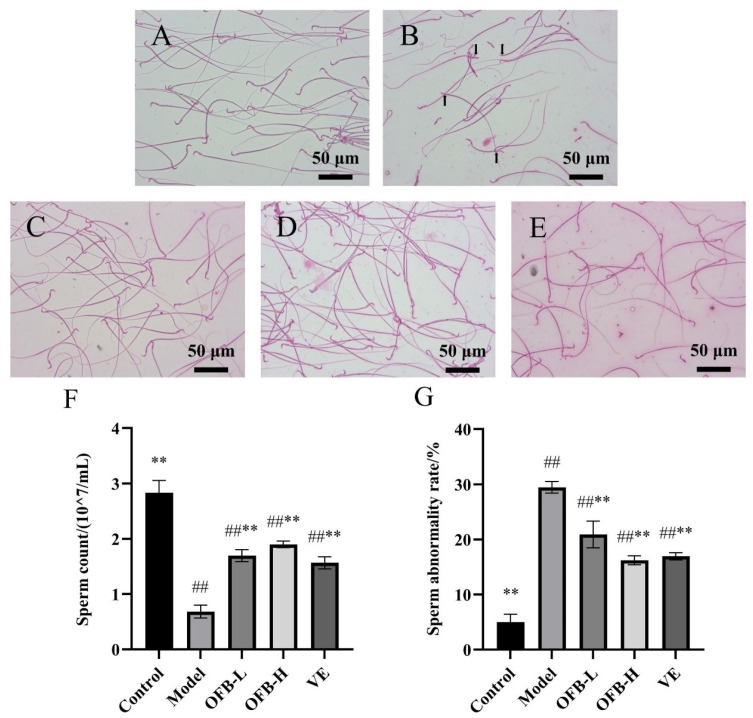
Comparison of sperm morphology, sperm count, and sperm abnormality rate among groups of rats (scale bar = 50 μm): (**A**) sperm morphology in the control group; (**B**) model group; (**C**) OFB-L group; (**D**) OFB-H group; (**E**) VE group. The black arrows indicate abnormal sperm. (**F**) Sperm count; (**G**) sperm abnormality rate. Symbol notation: # *p* < 0.05, ## *p* < 0.01 vs. control; * *p* < 0.05, ** *p* < 0.01 vs. model group.

Figure 2 illustrates the impact of OFB administration on sperm quality parameters. In the control group, sperm exhibited relatively intact morphology, clearly visible structures, and high sperm count (Figure 2A). Compared with the control group, the model group exhibited a large number of abnormal sperm, including headless, tailless, and folded-head sperm (Figure 2B). Sperm count was significantly decreased (*p* < 0.01), and the sperm abnormality rate was significantly increased (*p* < 0.01), indicating TG-induced sperm damage. Compared with the model group, both the OFB-L and OFB-H groups showed notable improvements in sperm morphology (Figure 2C,D). Sperm count was significantly elevated (*p* < 0.01), and the sperm abnormality rate was significantly reduced (*p* < 0.01). Ma et al. reported that zinc supplementation (ZnSO_4_) effectively improved sperm quality in male rats that were subjected to TG-induced reproductive toxicity, and the Zn-treated group showed notably better outcomes than the model group [25]. Consistent improvements were also observed in this study. These results suggested that OFB could alleviate TG-induced sperm abnormalities and improve sperm quality. Moreover, based on changes in sperm count, OFB showed even better restorative effects on sperm quality than the VE group.

### 2.5. Serum Sex Hormone Levels

**Figure 3:**
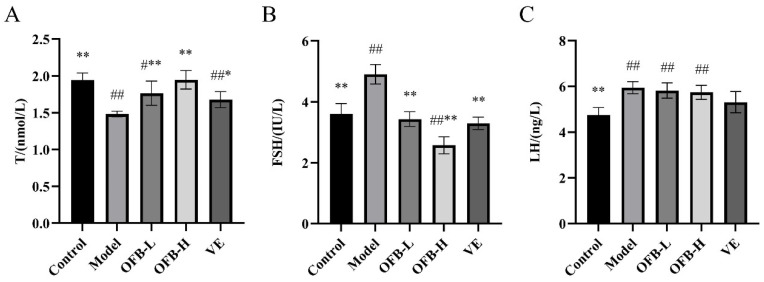
Comparison of serum sex hormone levels among groups of rats: (**A**) T levels; (**B**) FSH levels; (**C**) LH levels. Symbol notation: # *p* < 0.05, ## *p* < 0.01 vs. control; * *p* < 0.05, ** *p* < 0.01 vs. model group. T: testosterone; FSH: follicle-stimulating hormone; LH: luteinizing hormone.

As illustrated in Figure 3, compared with the control group, the model group exhibited substantially depressed T levels (*p* < 0.01), accompanied by significantly elevated FSH and LH levels (*p* < 0.01), suggesting TG-induced sex hormone disorder. Importantly, OFB administration dose-dependently restored hormonal balance, with both OFB-L and OFB-H groups demonstrating pronounced T level recovery (*p* < 0.01) and FSH suppression (*p* < 0.01) compared to the model group. Ma et al. reported that the dietary supplement DS could enhance testosterone synthesis and thereby restore TG-induced hormonal disorders in rats [26], a result that aligns with the outcomes observed in this study. These findings indicated that OFB intake could regulate TG-induced disturbances in sex hormone levels, thereby mitigating the adverse effects of TG. Furthermore, based on changes in T and FSH levels, the restorative effect of OFB on hormone regulation in damaged rats even exceeded that of the VE group.

### 2.6. Histopathological Analysis of Testicular Tissues

**Figure 4:**
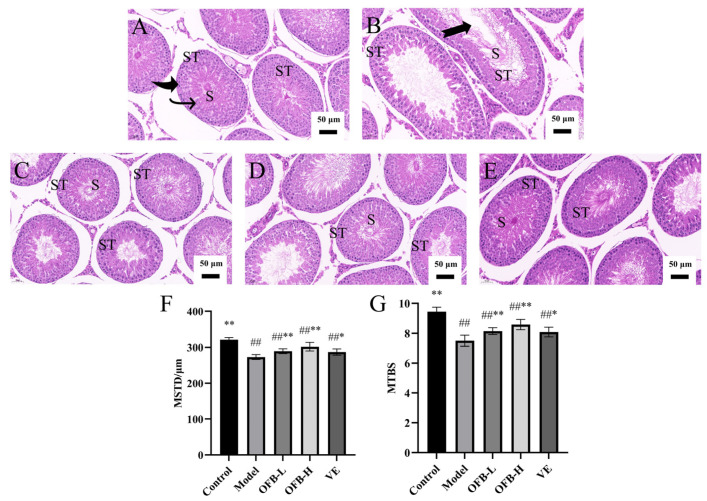
Histopathological examination of testicular tissues from each group (scale bar = 50 μm). Testicular histological sections stained with HE are shown for: (**A**) control group; (**B**) model group; (**C**) OFB-L group; (**D**) OFB-H group; (**E**) VE group. (**F**) Quantitative analysis of MSTD; (**G**) quantitative analysis of MTBS. Symbol notation: # *p* < 0.05, ## *p* < 0.01 vs. control; * *p* < 0.05, ** *p* < 0.01 vs. model group. ST: seminiferous tubules; S: sperm; thick arrows: spermatocytes; thin arrows: spermatids; swallowtail arrows: lumens; MSTD: mean seminiferous tubule diameter; MTBS: mean testicular biopsy score.

Figure 4 presents the histopathological changes in rat testicular tissues. In the model group (Figure 4B), the epithelial cell layer of the seminiferous tubules appeared thinner than that in the control group, accompanied by a marked reduction in spermatogenic cell numbers and sperm count. Additionally, the differentiation process from spermatogonia to mature sperm cells was poorly defined, and apparent vacuolation was observed within the seminiferous tubules. These pathological changes indicated that TG intake caused significant damage to the testicular tissue. Compared to the model group, OFB treatment notably increased the number of spermatogenic cells in the seminiferous tubules, and no apparent vacuolation was observed, as shown in Figure 4C,D. As shown in Figure 4F,G, both the mean seminiferous tubule diameter (MSTD) and mean testicular biopsy score (MTBS) were significantly decreased in the model group compared with the control group (*p* < 0.01). In contrast, MSTD and MTBS were significantly increased in the OFB-L and OFB-H groups compared with the model group (*p* < 0.01). These findings suggest that OFB effectively mitigated TG-induced histopathological damage in testicular tissues.

### 2.7. Oxidative Stress Markers in Testicular Tissue

**Figure 5:**
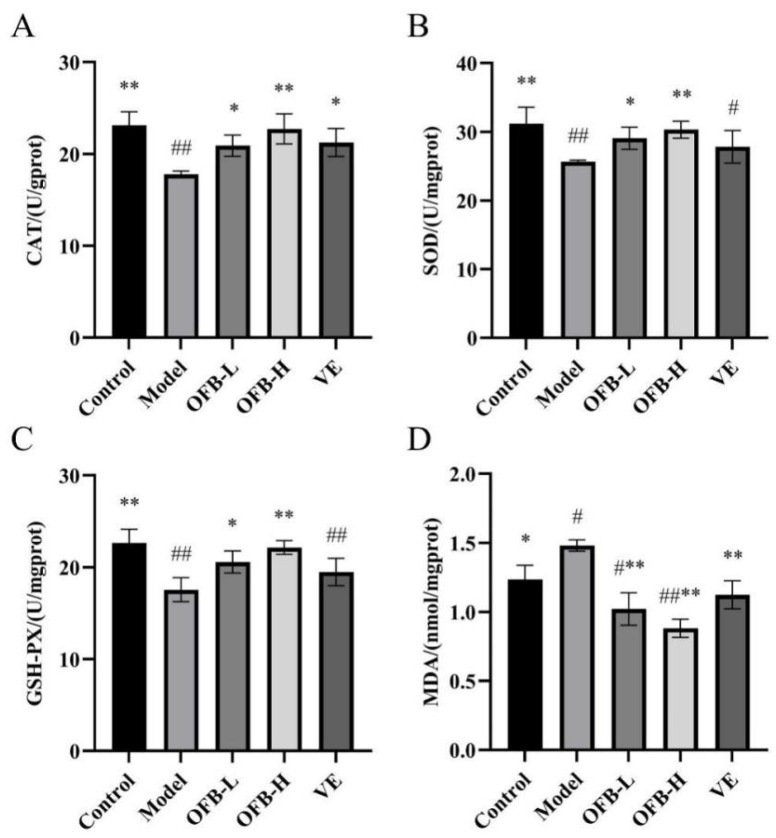
Comparison of oxidative stress markers in testicular tissues among groups of rats: (**A**) CAT activity; (**B**) SOD activity; (**C**) GSH-PX activity; (**D**) MDA levels. Symbol notation: # *p* < 0.05, ## *p* < 0.01 vs. control; * *p* < 0.05, ** *p* < 0.01 vs. model group. CAT: catalase; SOD: superoxide dismutase; GSH-PX: glutathione peroxidase; MDA: malondialdehyde.

As illustrated in Figure 5, compared with the control group, the model group exhibited a notable reduction in CAT, SOD, and GSH-PX activities (*p* < 0.01), concomitant with significantly increased MDA levels (*p* < 0.05), indicating that TG intake induced oxidative stress in testicular tissues. Relative to the model group, OFB administration elicited a dose-dependent enhancement of antioxidant enzyme activities. The OFB-L group demonstrated statistically significant elevations in CAT, SOD, and GSH-PX (*p* < 0.05), with the OFB-H group displaying more robust upregulation of these enzyme activities (*p* < 0.01). Moreover, both OFB-L and OFB-H groups exhibited extremely significant reductions in MDA levels (*p* < 0.01). Liu et al. reported that combined supplementation of zinc and selenium significantly improved SOD and MDA levels compared with the TG-induced model group and effectively alleviated oxidative damage in rats [27]. Collectively, these findings further confirmed the dose-responsive protective efficacy of OFB supplementation against TG-induced oxidative stress in testicular tissues. Moreover, based on the changes in CAT, SOD, GSH-PX, and MDA levels, the antioxidant recovery effects of OFB in damaged testicular tissue even surpassed those of the VE group.

## 3. Discussion

Many medications have the potential to negatively impact the male reproductive system and may even lead to infertility [20]. TG, a glycoside derived from the root of tripterygium wilfordii, and long-term administration of TG-containing drugs can readily cause damage to the male reproductive system [28]. The underlying mechanisms primarily involve oxidative stress, which may lead to spermatogenesis disorders, reproductive endocrine abnormalities, and germ cell apoptosis [29]. Due to these characteristics, TG has been widely employed in recent years for the establishment of animal models simulating male reproductive dysfunction [30,31,32]. In the present study, following TG administration, the model group exhibited testicular atrophy, increased sperm deformity, hormonal imbalances, vacuolation of seminiferous tubules, and oxidative stress in testicular tissue. These alterations align with earlier studies describing TG-induced reproductive injury [33], thereby validating the successful induction of a reproductive damage model in this study.

Fermentation is a microbial-driven metabolic process that serves as an effective approach to enhance the bioavailability of nutrients. Microbial fermentation typically involves a series of biochemical transformations that not only modify the composition of the substrate but also generate novel bioactive compounds. In this study, oyster homogenate was fermented with yeast using honey as the carbon source to produce OFB. Compared to the unfermented mixture, OFB showed decreased levels of reducing sugars and proteins (*p* < 0.05), while the composition of minerals, organic acids, amino acids, and phenolic compounds changed. The antioxidant capacity was also significantly enhanced (*p* < 0.05). Protein reduction may result from yeast breaking down oyster proteins into peptides and amino acids. The decrease in reducing sugars is likely due to their utilization by yeast for growth and metabolism, which also influences mineral content. The increase in organic acids is mainly due to yeast metabolism of amino acids and honey-derived sugars [34]. Amino acid profile changes are related to the enzymatic degradation of oyster proteins by yeast metabolism and the amino acid metabolic by-products during fermentation [35]. Additionally, the addition of honey enriched the content of sweet-tasting amino acids. Phenolic compounds mainly originate from honey, with some derived from amino acid metabolism. Their transformation is linked to yeast activity, including glycoside hydrolysis and phenolic acid degradation [36]. Overall, yeast fermentation altered the composition of active substances in OFB and enhanced its antioxidant activity, suggesting a potential strategy for improving reproductive health. Honey was selected due to its rich nutrients, fermentable sugars, and antibacterial properties. However, given its allergenic potential, future studies could explore simpler, low-allergen alternatives (e.g., maple syrup or sorghum syrup) that provide fermentable sugars, though they may lack honey’s natural polyphenols and antioxidants.

The composition of the OFB was analyzed, and the results showed that OFB contains various active substances such as taurine, proteins, zinc, free amino acids, organic acids, polyphenols, and flavonoids. Numerous studies have demonstrated that foods containing minerals (zinc and selenium), arginine, flavonoids, peptides, and other bioactive substances can improve sperm quality and reproductive function [37,38,39,40]. For example, Owumi et al. reported that taurine exerted protective effects against BaP-induced reproductive toxicity in rats by mitigating oxidative stress and inflammatory responses, which in turn enhanced sperm parameters and normalized hormone profiles [41]. Zhang et al. reported that arginine could counteract T-2 toxin-induced reproductive toxicity by enhancing sperm parameters and increasing testosterone levels [42]. Santos et al. highlighted the key role of zinc in maintaining sperm morphology and function [43]. Other studies have shown that substances produced from oysters through microbial fermentation, such as oyster peptides, can alleviate oxidative stress damage in the body. For instance, Oh et al. reported that extracts from fermented oysters could enhance antioxidant defenses by promoting Nrf2/HO-1 pathway activation [44]. Li et al. found that oyster peptides can increase androgen levels, improve the pathological state of testicular tissue, and alleviate oxidative stress, demonstrating beneficial effects on reproductive damage [45]. These results offer substantial theoretical evidence supporting the potential reproductive-enhancing effects of OFB. Based on this, we hypothesized that OFB might have a protective effect on reproductive damage. Subsequently, we found both low- (5 mL/kg, equivalent to 60 mL/day for human) and high-dose (10 mL/kg, equivalent to 120 mL/day for human) OFB administration alleviated TG-induced reproductive damage in rats and a notable dose–effect relationship was observed. This might be attributed to the reproductive-health-protective effects of various bioactive components present in OFB. The bioavailability of peptides and zinc is generally low when taken orally. However, small molecules like taurine and reducing sugars are more easily absorbed, and free amino acids are absorbed faster than whole proteins. Polyphenols and flavonoids usually have low bioavailability due to poor solubility and first-pass metabolism. Studies have shown that oyster peptide-zinc complexes are better absorbed and may improve zinc bioavailability [46]. Therefore, zinc in OFB may be more bioavailable when bound to peptides. Organic acids in OFB might also help absorption by forming complexes with metal ions or polyphenols [47].

The weight of reproductive organs, including the testes and prostate, is closely linked to the structural and physiological state of the reproductive system and serves as an indicator of tissue pathology. Spermatogenesis is carried out within the seminiferous tubules, where the preservation of testicular structure and function is critical for sperm production [48]. Minor changes, such as a decrease in seminiferous tubule atrophy and thinning of the germinal epithelium, directly reflect a decrease in testicular weight. Combined with histopathological examination results, the harmful or protective effects of drugs on reproductive organs can be evaluated. Our findings showed that TG significantly reduced the organ indices and sperm quality (*p* < 0.01), whereas OFB intervention effectively mitigated these effects by significantly increasing the testicular index, seminal vesicle + prostate index, spleen index, and sperm count (*p* < 0.01). Histopathological examinations further confirmed that OFB enhanced the quantity of both interstitial and spermatogenic cells, decreased the seminiferous tubule lumen area, and improved both MSTD and MTBS values in testicular tissue, suggesting its role in alleviating TG-induced testicular histopathological damage. These observations align with those previously reported by Ma et al. [25] and Karagüzel et al. [49]. Thus, we speculated that OFB might restore reproductive organ indices and sperm quality by repairing TG-induced testicular tissue damage.

The hypothalamus–pituitary–gonadal (HPG) axis is crucial for regulating male reproductive physiology, primarily by controlling testosterone production in the testes [50]. Gonadotropin-releasing hormone (GnRH) secreted by the hypothalamus stimulates the anterior pituitary to release FSH and LH. FSH, in turn, acts primarily on Sertoli cells in the testes, supporting spermatogenesis and increasing the synthesis of androgen-binding protein (ABP), which helps maintain high testosterone levels locally within the testes. Concurrently, LH targets Leydig cells to stimulate the biosynthesis of testosterone, a critical androgen involved in the development and sustenance of male reproductive capacity. Testosterone further exerts a negative feedback effect on both the hypothalamus and pituitary gland, thereby ensuring the stability of the HPG axis and maintaining endocrine homeostasis [51]. Our results demonstrated that TG significantly decreased serum testosterone levels while increasing FSH and LH levels (*p* < 0.01), indicating a disruption of the endocrine balance. Following OFB intervention, testosterone levels was significantly increased (*p* < 0.01), while FSH and LH levels tended to normalize. These findings are in line with the regulatory function of the HPG axis [52]. Wang et al. reported that oyster hydrolysates could significantly promote the synthesis of sex hormones in hemi-castrated rats [53]. He et al. found that reduced testosterone triggers negative feedback on the HPG axis, increasing FSH and LH levels [54]. In our study, we observed a comparable pattern. Therefore, we speculate that OFB may also regulate sex hormones through the negative feedback mechanism of the HPG axis [55]. Additionally, TG exposure led to lower testosterone levels and reduced sperm count, likely due to Leydig cell damage. OFB treatment alleviated these effects, indicating it may help restore testosterone levels by protecting Leydig cells.

Oxidative stress plays a pivotal role in impairing male reproductive function, leading to impaired spermatogenesis through apoptosis of Leydig cells, Sertoli cells, and germ cells [56]. In our study, the model group exhibited a substantial decline in testicular antioxidant capacity, as evidenced by significantly depressed CAT, SOD, and GSH-PX activities (*p* < 0.01), coupled with markedly elevated MDA accumulation (*p* < 0.05), collectively suggesting TG-induced testicular oxidative impairment. Notably, OFB intervention effectively attenuated these alterations, restoring antioxidant enzyme activities (*p* < 0.05) while concurrently reducing lipid peroxidation as indicated by decreased MDA levels (*p* < 0.05). These results were in line with the in vitro antioxidant test. Shafie et al. reported that Cocos nucifera L. water improved reproductive health by enhancing antioxidant defense mechanisms, thereby alleviating BPA-induced reproductive damage [57]. Similarly, Fu et al. demonstrated that GHYSJ provided notable protective effects against diabetes-induced male reproductive injury through the modulation of oxidative stress pathways [58]. These studies support the notion that oxidative stress is a critical regulatory mechanism in male reproductive damage and that mitigating oxidative stress can alleviate such injuries. The study by Liu et al. demonstrated that fermented oyster hydrolysate possesses the ability to alleviate oxidative stress, reduce the expression of pro-inflammatory cytokines, and enhance the levels of protective factors [23], which is consistent with our findings. Collectively, these results suggest that OFB might improve reproductive function through exerting antioxidative properties. Although our findings demonstrate the antioxidant potential of OFB, the precise molecular mechanisms underlying its effects remain to be fully elucidated. Previous studies have shown that oyster peptides can activate the Nrf2, HO-1, and NQO1 signaling pathways [7]; polyphenolic compounds can inhibit apoptosis via the PI3K/Akt pathway [59]; and zinc is associated with the activation of the Nrf2 pathway [27]. Therefore, we speculate that OFB may integrate these pathways and play a key role in modulating oxidative stress.

## 4. Materials and Methods

### 4.1. Materials and Reagents

OFB was prepared in the laboratory. *Saccharomyces cerevisiae* strain BV818 active dry yeast was obtained from Angel Yeast Co., Ltd. (Yichang, China). Tripterygium glycosides (TG) tablets (10 mg/tablet) were supplied by Hunan Qianjin Xieli Pharmaceutical Co., Ltd. (Zhuzhou, China), and vitamin E (VE, 50 mg/tablet) was provided by Guangzhou Baiyunshan Xingqun Pharmaceutical Co., Ltd. (Guangzhou, China). ELISA kits for rat testosterone (T), follicle-stimulating hormone (FSH), and luteinizing hormone (LH) were sourced from Jiangsu Meimian Industrial Co., Ltd. (Yancheng, China). Assay kits for total antioxidant capacity, catalase (CAT), superoxide dismutase (SOD), glutathione peroxidase (GSH-PX), and malondialdehyde (MDA) were purchased from Nanjing Jiancheng Bioengineering Institute (Nanjing, China). All other reagents used in this study were of analytical grade.

### 4.2. Instruments and Equipment

The main experimental instruments employed in this study comprised the LTN2-16K desktop high-speed centrifuge (Qingdao Lantern Scientific Instrument Equipment Co., Ltd., Qingdao, China), PHS-3E pH meter (Shanghai INESA Scientific Instrument Co., Ltd., Shanghai, China), CBCL-24 cryogenic grinder (Shanghai Cebo Biotechnology Development Center, Shanghai, China), UV-8000 UV-visible spectrophotometer (Shanghai Metash Instruments Co., Ltd., Shanghai, China), Spark multifunctional microplate reader (Tecan Trading, Männedorf, Switzerland), and NLCD-500 digital biological microscope (Nanjing Jiangnan Novel Optics Co., Ltd., Nanjing, China).

### 4.3. Experimental Animals

Forty-eight 6-week-old male SD rats (body weight 180–220 g) were randomly allocated to six experimental groups (*n* = 8 per group). The animals were maintained at 21 ± 2 °C with 50 ± 10% relative humidity in a controlled environment, with ad libitum access to standard rodent diet and water. All experimental protocols were conducted in accordance with the ethical guidelines approved by Qingdao University Animal Ethics Committee (Approval No. QDU-AEC-2024442).

### 4.4. OFB Preparation Process

Fresh oysters were shelled, washed, and steamed at 80 °C for 3 min. After homogenization, purified water was added at a 1:3 (*w*/*v*) ratio. Subsequently, honey was incorporated at 6% (*w*/*v*), and the pH of the mixture was adjusted to 4.5 prior to sterilization. After cooling to 25 °C, 2 g/L BV818 yeast was inoculated, and fermentation was conducted under static conditions at 28 °C for 7 days. The fermentation broth was then centrifuged and filtered to obtain the clarified OFB.

### 4.5. Experimental Methods

#### 4.5.1. Determination of Physicochemical Properties, Nutritional Components, and Antioxidant Capacity of OFB

(1) Physicochemical analysis of OFB

The alcohol content of OFB was assessed using the alcohol meter method, and the pH was recorded with a digital pH meter. Total acidity was assessed by acid-base titration according to the Chinese National Food Safety Standard GB 12456-2021.

The reducing sugar content was measured using the DNS colorimetric method. A 1 mL sample was mixed with 1 mL distilled water and 1.5 mL DNS reagent, heated in boiling water for 5 min, cooled, and diluted to 25 mL. Absorbance was read at 540 nm. A standard curve (r^2^ = 0.999) was prepared using serial dilutions of a 1 mg/mL glucose solution, and reducing sugar content was calculated accordingly. The formula was as follows:

(1) $$Reducing\;sugar\;content\;(mg/mL)=\frac{Mass\;of\;reducing\;sugar\times Dilution\;factor}{Sample\;volume}$$

The reducing sugar composition was analyzed by high-performance liquid chromatography with a refractive index detector (HPLC-RID) (Agilent 1260; Agilent Technologies, Santa Clara, CA, USA) after filtration through a 0.45 μm membrane. Glucose, galactose, fructose, and hydrolyzed glucose were separated on an Xtimate^®^ Sugar-Ca column (7.8 × 300 mm, 6 μm; Welch Materials, Shanghai, China) using water as the mobile phase (1.0 mL/min, 20 μL injection, 80 °C). Maltose and lactose were separated on an Agilent amino column (4.6 × 250 mm, 5 μm; Agilent Technologies, Santa Clara, CA, USA) with an acetonitrile/water (70:30, *v*/*v*) mobile phase (1.0 mL/min, 10 μL injection, 35 °C).

(2) Analysis of bioactive components in OFB

Taurine and amino acids were determined by high-performance liquid chromatography (HPLC) after derivatization with dansyl chloride. Samples were diluted, reacted at 60 °C for 30 min in the dark, and stopped with methylamine hydrochloride. After filtration (0.22 μm), the products were analyzed using an Agilent 1260 HPLC system (Agilent Technologies, Santa Clara, CA, USA) with a Diamonsil C18 column (4.6 × 250 mm, 5 μm; Dikma Technologies, Beijing, China). The mobile phase consisted of sodium acetate buffer (10 mM, pH 4.2) and acetonitrile (70:30, *v*/*v*), with a flow rate of 1.0 mL/min at 40 °C, detection at 254 nm, and 20 μL injection volume. Quantification was based on a standard calibration curve (r^2^ = 0.999).

Organic acids were analyzed using a Shimadzu LC-20AD HPLC system (Shimadzu Corp., Kyoto, Japan) with an Ultimate AQ-C18 column (4.6 × 250 mm, 5 μm; Welch Materials, Shanghai, China) after filtration (0.22 μm). The mobile phase consisted of disodium hydrogen phosphate (20 mM, pH 2.6) and methanol (95:5, *v*/*v*), with a flow rate of 0.7 mL/min. The column was maintained at 30 °C, with detection at 210 nm, injection volume of 10 μL, and a total runtime of 35 min. Organic acids were identified and quantified by external standards.

Mineral elements (Zn, K, Na, Ca, and P) were determined using an iCAP RQ ICP-MS (Thermo Fisher Scientific, Waltham, MA, USA) after microwave digestion. Briefly, 0.5 mL of sample was digested with 10 mL of 67% HNO_3_ in a Teflon vessel at 200 °C using a Mars 5 system (CEM Corp., Matthews, NC, USA) and diluted to 25 mL. Calibration was performed using multi-element standards in 1% HNO_3_.

Total phenolic content was determined by the Folin–Ciocalteu method [60]. A 0.8 mL sample was mixed with distilled water and Folin–Ciocalteu reagent, left in the dark for 5 min, then reacted with 12% sodium carbonate. After dilution to 25 mL, the mixture was incubated for 90 min in the dark. Absorbance was measured at 765 nm. Gallic acid was used as a standard (r^2^ = 0.990), and results were expressed as gallic acid equivalents.

Total flavonoid content was measured following Neupane and Lamichhane’s method [61]. A 5 mL sample was mixed with sodium nitrite, aluminum nitrate, and sodium hydroxide solutions, and then diluted to 10 mL and left to stand for 15 min. Absorbance was recorded at 510 nm. Rutin was used as the standard (r^2^ = 0.999), and results were expressed as rutin equivalents.

(3) In vitro antioxidant activity assay of OFB

The total antioxidant capacity was determined according to the instructions of the kit.

The DPPH radical scavenging activity was determined with slight modifications based on the method of Bibi et al. [62]. A 0.2 mmol/L DPPH solution was mixed with the sample (1:1, *v*/*v*) and incubated in the dark for 30 min. Absorbance was measured at 517 nm (A1). The absorbance values measured with ethanol substitute samples and DPPH were A2 and A3, respectively. The formula was as follows:

(2) $$DPPH\;radical\;scavenging\;rate\left(\%\right)=\left(1-\frac{A1-A2}{A3}\right)\times 100\%$$

The ABTS radical scavenging activity was determined with slight modifications according to the method described by Asem et al. [63]. ABTS working solution and sample (0.8 mL + 0.2 mL) were mixed and left in the dark for 6 min. Absorbance at 734 nm was recorded as B1 (ABTS+ sample), B2 (ethanol + sample), and B3 (ABTS + ethanol). The formula was as follows:

(3) $$ABTS\;radical\;scavenging\;rate\left(\%\right)=\left(1-\frac{B1-B2}{B3}\right)\times 100\%$$

The hydroxyl radical scavenging activity was determined with slight modifications based on the method described by Lan et al. [64]. A mixture of 9 mmol/L ethanol–salicylic acid solution, sample, 9 mmol/L ferrous sulfate, and 8.8 mmol/L hydrogen peroxide was incubated at 37 °C in the dark for 30 min. Absorbance at 510 nm was recorded as C1. Controls with distilled water replacing hydrogen peroxide or sample gave absorbance values C2 and C3. The formula was as follows:

(4) $$Hydroxyl\;radical\;scavenging\;rate\left(\%\right)=\left(1-\frac{C1-C2}{C3}\right)\times 100$$

(4) Ultra-high-performance liquid chromatography–tandem mass spectrometry (UHPLC-MS/MS) Analysis

Phenolic compounds were analyzed using UHPLC-MS/MS (Thermo Vanquish UHPLC with Q-Exactive HF MS; Thermo Fisher Scientific, Waltham, MA, USA). Separation was performed on a Zorbax Eclipse C18 column (2.1 × 100 mm, 1.8 μm; Agilent Technologies, Santa Clara, CA, USA) at 30 °C, with a 0.3 mL/min flow rate and a 2 μL injection volume. The mobile phase was 0.1% formic acid in water (A) and acetonitrile (B), using a gradient elution. Mass spectrometry was performed in both positive and negative ion modes using dynamic multiple reaction monitoring (MRM). Key parameters included a spray voltage of 3.5 kV, capillary temperature of 330 °C, heater temperature of 325 °C, sheath/auxiliary gas flow rates of 45/15 (arb), and S-Lens RF level of 55%. Data were analyzed with Compound Discoverer 3.3 and identified by comparing MS/MS spectra to mzCloud and mzVault. Relative contents were calculated using 2-chlorophenylalanine (1.0 mg/L) as the internal standard.

#### 4.5.2. Animal Grouping and Administration

Rats were randomly assigned into six groups: control group (an equivalent volume of saline), model group (30 mg/kg TG + saline), OFB-L group (30 mg/kg TG + 5 mL/kg OFB), OFB-H group (30 mg/kg TG + 10 mL/kg OFB), and VE group (30 mg/kg TG + 6.50 mg/kg VE). As shown in Figure S1, all rats were housed under standard conditions for 8 weeks. The control group received saline throughout. The other groups were first given TG (30 mg/kg) for 4 weeks to induce reproductive dysfunction. In the following 4 weeks, the model group received saline, while the OFB-L and OFB-H groups were administered OFB at doses of 5 mL/kg and 10 mL/kg, respectively, by oral gavage. The VE group was administered VE at a dose of 6.50 mg/kg via gavage. The OFB dosing regimen (5 mL/kg for the OFB-L group and 10 mL/kg for the OFB-H group) was determined based on preliminary toxicity tests, established safety ranges, and effective doses reported in the study by Wang et al. [65].

#### 4.5.3. Blood Collection and Analysis

Peripheral blood samples (1 mL) were subjected to complete hematological profiling using an automated analyzer to quantify white blood cell (WBC) count, red blood cell (RBC) count, hemoglobin (HGB) concentration, lymphocyte percentage (LYM), and neutrophil percentage (NEU). After the initial analysis, the remaining blood samples were centrifuged (3000× *g*, 15 min) to separate the serum, which was subsequently stored at −80 °C for subsequent biochemical assays.

#### 4.5.4. Organ Index Determination

The weights of testes, epididymides, prostate + seminal vesicles, kidneys, spleens and livers were measured to determine organ indices, calculated as:

(5) $$Organ\;index\;(mg/g)\;=\frac{Organ\;weight}{Body\;weight}$$

#### 4.5.5. Sperm Quality Assessment

One epididymis was placed in 1 mL of HTF medium at 37 °C, minced, and incubated for 5 min to facilitate sperm release and dispersion. A hemocytometer was used to calculate the sperm count following the equation (where N represents the count value of five small squares):

(6) $$Sper\;mcount\;(million/mL)\;=N\times 5\times Dilution\;factor\times 10^{4}$$

Sperm smears were prepared after eosin staining, observed under a microscope, and the number of abnormal sperm was recorded. The sperm abnormality rate was calculated using the following equation:

(7) $$Sperm\;abnormality\;rate\left(\%\right)=\frac{Number\;of\;abnormal\;sperm}{Total\;evaluated\;sperm}\times 100\%$$

#### 4.5.6. Determination of Serum Sex Hormone Levels

Serum concentrations of T, FSH, and LH were quantified using commercially available ELISA kits, in accordance with the manufacturer’s instructions.

#### 4.5.7. Oxidative Stress Index Assays in Testicular Tissue

Testicular tissues were homogenized in ice-cold saline (1:9 *w*/*v*) and centrifuged (3000× *g*, 10 min, 4 °C) to obtain the supernatant fraction. The resulting supernatant was subsequently analyzed for MDA content and antioxidant enzyme activities, including CAT, SOD, and GSH-PX, using commercial assay kits according to the manufacturer’s specifications.

#### 4.5.8. Histological Observation

The left testes were immersed in fixative for 48 h, dehydrated, embedded in paraffin, sectioned, dewaxed, rehydrated, and stained. Sections were examined and imaged under a microscope. The MSTD was determined by randomly selecting 20 relatively round seminiferous tubules per section using CaseViewer 2.4.0 software (3DHISTECH, Budapest, Hungary) and calculating the average diameter [66]. The MTBS was obtained by scoring the seminiferous tubules based on the Johnsen scoring system and calculating the average value [67].

#### 4.5.9. Statistical Analysis

Statistical analyses were performed with SPSS 26.0 (IBM Corp., Armonk, NY, USA), and graphs were generated using GraphPad Prism 9.5 (GraphPad Software Inc., San Diego, CA, USA). Data are presented as mean ± standard deviation (S.D.). Group comparisons were conducted by one-way ANOVA with Tukey’s post hoc test, with statistical significance thresholds set at *p* < 0.05 and *p* < 0.01.

## 5. Conclusions

In this study, oysters were fermented with yeast to prepare OFB, which is rich in various bioactive compounds such as taurine, zinc, amino acids, and phenolic acids. OFB administration effectively ameliorated TG-induced reproductive injury in rats. Potential mechanisms were related to the antioxidant activity of OFB. These results offer valuable insights for the formulation of functional beverages utilizing oyster resources. However, the precise molecular mechanisms underlying the ameliorative effects of OFB remain to be fully elucidated. Future studies should focus on isolating and purifying key active components or integrating metabolomics approaches and apoptosis marker analysis to gain deeper insights into the mechanisms of action.

## Figures and Tables

**Table 1:** Component characterization of OFB.

Test Parameters	Test Indicators	Pre-Fermentation	OFB
Physicochemical properties	Total acidity/(g/L)	1.36 ± 0.07 ^b^	4.27 ± 0.19 ^a^
	Reducing sugars/(g/L)	60.64 ± 0.81 ^a^	2.54 ± 0.14 ^b^
	Protein content/(g/L)	1.59 ± 1.86 ^a^	1.19 ± 1.15 ^b^
	Taurine/(g/L)	0.74 ± 0.03 ^a^	0.76 ± 0.03 ^a^
Reducing sugar composition	Glucose/(g/L)	19.73	0.28
	Fructose/(g/L)	34.17	2.12
	Hydrolyzed glucose/(g/L)	9.13	0.93
Mineral element content/(mg/L)	Zn^2+^	0.70	1.10
	K^+^	60.00	71.00
	Na^+^	25.00	31.00
	Ca^2+^	7.00	9.00
	P^5+^	17.00	9.00
	Total	109.70	121.10
Organic acid content/(μg/mL)	Oxalic acid	13.19	0.23
	Malic acid	4.22	445.86
	Lactic acid	11.96	272.33
	Fumaric acid	0.13	4.37
	Succinic acid	100.82	1528.30
	Citric acid	13.02	52.56
	Total	143.34	2303.66
Amino acid content/(mg/L)	Lysine *	6.11	20.72
	Phenylalanine *	5.39	7.80
	Methionine *	1.45	1.64
	Threonine *	17.12	6.30
	Isoleucine *	15.13	29.30
	Leucine *	32.31	53.50
	Valine *	8.73	18.00
	Histidine *	4.16	7.80
	Arginine *	39.25	41.92
	Serine	5.53	14.20
	Glycine	14.54	18.10
	Aspartic acid	60.16	94.54
	Glutamic acid	76.94	89.70
	Alanine	14.35	13.20
	Proline	20.35	5.20
	Tyrosine	2.55	5.41
	Cystine	5.54	6.87
	Essential amino acids	129.65	186.98
	Non-essential amino acids	199.96	247.22
	Total	329.61	434.20

Note: Different superscript letters denote statistically significant differences (*p* < 0.05), while shared letters indicate no significant variation (*p* > 0.05) between treatments; “*” indicates essential amino acids. OFB: oyster fermentation broth.

**Table 2:** In vitro antioxidant activity of OFB.

Test Parameters	Pre-Fermentation	OFB
Polyphenol/(mg/L GAE)	89.42 ± 5.91 ^b^	123.00 ± 1.46 ^a^
Flavone/(mg/L RE)	1.19 ± 0.14 ^b^	1.97 ± 0.17 ^a^
Total antioxidant capacity/(U/mL)	0.86 ± 0.13 ^b^	1.28 ± 0.09 ^a^
DPPH scavenging activity/%	41.20 ± 3.72 ^b^	55.80 ± 0.68 ^a^
ABTS scavenging activity/%	54.95 ± 6.60 ^b^	69.54 ± 2.35 ^a^
OH- scavenging activity/%	14.77 ± 1.74 ^b^	48.36 ± 1.20 ^a^

Note: Gallic acid equivalent and Rutin equivalent are denoted as GAE and RE. Different superscript letters denote statistically significant differences (*p* < 0.05), while shared letters indicate no significant variation (*p* > 0.05) between treatments.

**Table 3:** Phenolic compound profile of OFB.

Phenolic Compounds	RT	Formula	CAS	Content/(mg/L)	
				Pre-Fermentation	OFB
3,4-dihydroxyphenylpyruvic acid	0.756	C_9_H_8_O_5_	4228-66-4	ND	0.98
Caffeic acid	0.803	C_9_H_8_O_4_	331-39-5	1.74	0.18
Pyrogallol	1.344	C_6_H_6_O_3_	87-66-1	ND	1.44
Xanthurenic acid	4.722	C_10_H_7_NO_4_	59-00-7	3.20	1.16
Clove3	5.054	C_16_H_18_O_9_	152041-16-2	0.39	ND
Salicylic acid	5.216	C_7_H_6_O_3_	69-72-7	ND	1.44
Isoliquiritigenin	5.624	C_15_H_12_O_4_	961-29-5	ND	0.03
Robinin	6.094	C_33_H_40_O_19_	301-19-9	0.04	0.02
4-Hydroxybenzaldehyde	6.121	C_7_H_6_O_2_	123-08-0	ND	0.40
Quercetin	9.104	C_15_H_10_O_7_	117-39-5	7.07	4.15
Naringenin	9.988	C_15_H_12_O_5_	480-41-1	ND	0.32

Note: RT: retention time; ND: not detected.

**Table 4:** Comparison of hematological parameters among groups of rats.

Groups	WBC/(10^9^/L)	RBC/(10^12^/L)	HGB/(g/L)	LYM/%	NEU/%
Control	7.45 ± 0.34	7.44 ± 0.61	151.80 ± 5.89	70.62 ± 2.99 **	22.67 ± 1.10 *
Model	7.41 ± 0.42	7.23 ± 0.53	150.20 ± 5.85	66.87 ± 2.16 ##	19.80 ± 1.87 #
OFB-L	7.68 ± 0.34	8.05 ± 0.49 #**	154.00 ± 3.46	71.46 ± 1.23 **	19.43 ± 0.98 ##
OFB-M	7.81 ± 0.30	8.32 ± 0.32 ##**	164.60 ± 5.77 #**	72.12 ± 0.85 **	22.73 ± 1.18 *
VE	8.24 ± 0.35 ##**	7.86 ± 0.30 *	149.20 ± 4.82	70.42 ± 1.24 **	22.57 ± 1.18 *

Note: Symbol notation: # *p* < 0.05, ## *p* < 0.01 vs. control; * *p* < 0.05, ** *p* < 0.01 vs. model group. OFB-L/H: low/high-dose oyster fermentation broth; VE: vitamin E; WBC: white blood cell; RBC: red blood cell; HGB: hemoglobin; LYM: lymphocyte; NEU: neutrophil percentage.

## Data Availability

The data that supports the findings of this study are included in this published article.

## Supplementary Materials

Supporting information can be downloaded at: https://www.mdpi.com/article/10.3390/molecules30173550/s1. Table S1. Retention time of the main components of OFB. Figure S1. The structure of phenolic compounds. Figure S2. Animal experimental administration methods.

## Abbreviations

The following abbreviations are used in this manuscript:

OFB	Oyster fermentation broth
TG	Tripterygium glycosides
VE	Vitamin E
T	Testosterone
FSH	Follicle-stimulating hormone
LH	Luteinizing hormone
CAT	Catalase
SOD	Superoxide dismutase
GSH-PX	Glutathione peroxidase
MDA	Malondialdehyde
OFB-L	Oyster fermentation broth low dose
OFB-H	Oyster fermentation broth high dose
MSTD	Mean seminiferous tubule diameter
MTBS	Mean testicular biopsy score
HPLC	High-performance liquid chromatography
ICP-MS	Inductively coupled plasma–mass spectrometry
WBC	White blood cell
RBC	Red blood cell
HGB	Hemoglobin
LYM	Lymphocyte
NEU	Neutrophil
HPG	Hypothalamus–pituitary–gonadal
GnRH	Gonadotropin-releasing hormone
ABP	Androgen-binding protein
