# Supplementary material for: Oyster Fermentation Broth Alleviated Tripterygium-Glycosides-Induced Reproductive Damage in Male Rats
Source: Molecules. 2025 Aug 29;30(17):3550. doi: 10.3390/molecules30173550 (PMC12430144; doi:10.3390/molecules30173550)
Supplement: Supplementary file 1 [file molecules-30-03550-s001.zip › molecules-3725794-supplementary.pdf]

Table S1. Retention time of the main components of OFB.

Component	Retain time/ min	
	Pre-fermentation	OFB
Taurine	6.941	6.934
Glucose	11.610	11.601
Fructose	13.731	13.722
Hydrolyzed glucose	11.610	11.601
Oxalic acid	4.346	4.428
Malic acid	6.574	6.550
Lactic acid	8.057	7.993
Fumaric acid	13.404	13.301
Succinic acid	14.216	13.968
Citric acid	11.738	11.777
Lysine	39.232	39.412
Phenylalanine	37.256	37.238
Methionine	32.704	32.707
Threonine	21.989	21.986
Isoleucine	34.823	34.813
Leucine	35.168	35.156
Valine	31.402	31.393
Histidine	18.896	18.895
Arginine	21.582	21.578
Serine	15.582	15.576
Glycine	16.710	16.698
Aspartic acid	7.341	7.358
Glutamic acid	8.532	8.542
Alanine	22.931	22.924
Proline	23.566	23.548
Tyrosine	30.492	30.385
Cystine	35.549	35.757

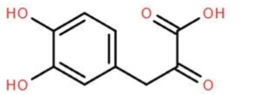

3,4-dihydroxyphenylpyruvic acid

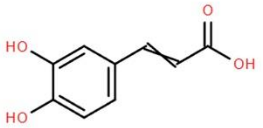

Caffeic acid

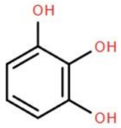

Pyrogallol

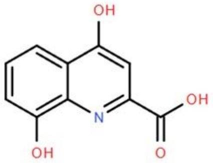

Xanthurenic acid

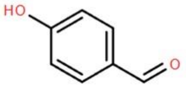

4-Hydroxybenzaldehyde

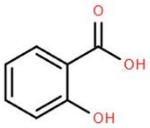

Salicylic acid

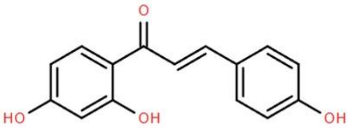

Isoliquiritigenin

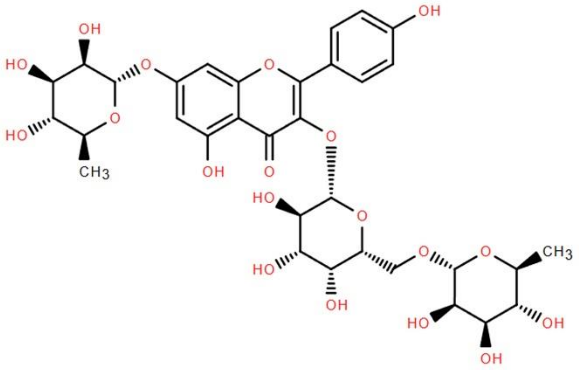

Robinin

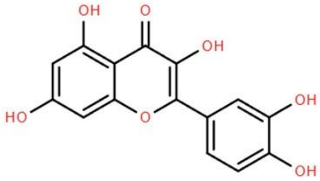

Quercetin

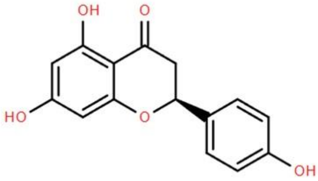

Naringenin

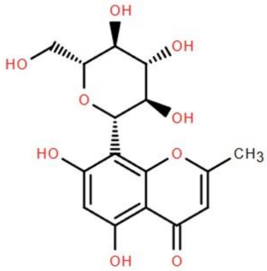

Clove3

Figure S1. The structure of phenolic compounds.

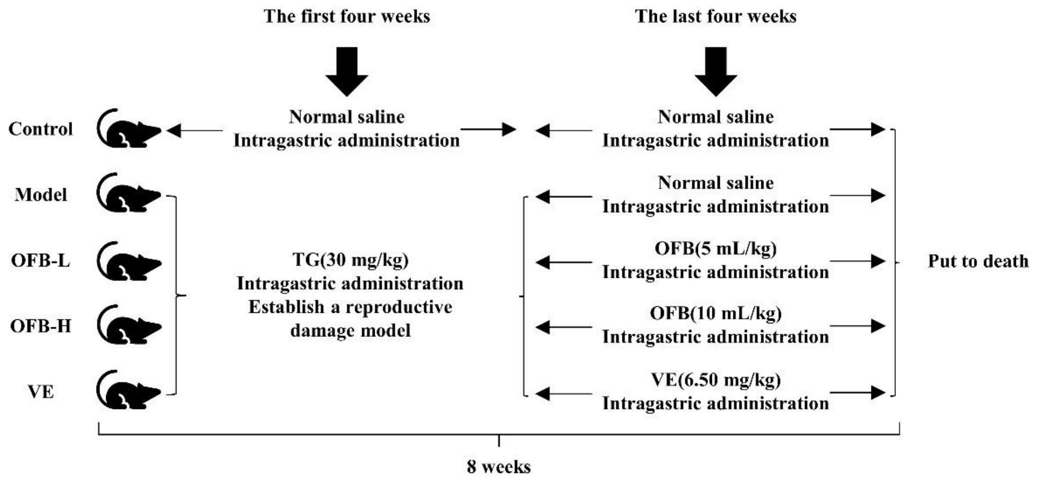

Figure S2. Animal experimental administration methods.
